# The Persistent Sodium Current Blocker Riluzole Is Antiarrhythmic and Anti-Ischaemic in a Pig Model of Acute Myocardial Infarction

**DOI:** 10.1371/journal.pone.0014103

**Published:** 2010-11-24

**Authors:** Steven M. Weiss, David A. Saint

**Affiliations:** 1 Australian National University, Canberra, Australia; 2 University of Adelaide, Adelaide, Australia; Maastricht University, Netherlands

## Abstract

**Background:**

The potential of the cardiac persistent sodium current as a target for protection of the myocardium from ischaemia and reperfusion injury is gaining increasing interest. We have investigated the anti-ischaemic and antiarrhythmic effects of riluzole, a selective INaP blocker, in an open chest pig model of infarction.

**Methods and Principal Findings:**

The left anterior descending coronary artery (LAD) was ligated in 27 anesthetised pigs (landrace or large white, either sex, 20–35 kg) which had received riluzole (8 mg/kg IP; n = 6), lidocaine (2.5–12 mg/kg bolus plus 0.05–0.24 mg/kg/min; n = 11) or vehicle (n = 10) 50 min prior. Arrhythmias could be delineated into phase 1a (0 to 20 min), phase 1b (20 to 50 min) and phase 2 (from 50 min to termination at 180 min) and were classified as premature ventricular contractions (PVCs), non-sustained ventricular tachycardia (VT) or ventricular fibrillation (VF) (spontaneously reverting within 15 s) or sustained VT or VF (ie. requiring cardioversion at 15 s). Riluzole reduced the average number of all arrhythmias in Phase 2 (PVCs from 484+/−119 to 32+/−13; non sustained arrhythmias from 8.9+/−4.4 to 0.7+/−0.5; sustained arrhythmias from 3.9+/−2.2 to 0.5+/−0.4); lidocaine reduced the average number of non-sustained and sustained arrhythmias (to 0.4+/−0.3 and 0.4+/−0.3 respectively) but not PVCs (to 390+/−234). Riluzole and lidocaine reduced the average number of sustained arrhythmias in phase 1b (from 1.8+/−0.4 to 0.17+/−0.13 (p<0.02) and to 0.55+/−0.26 (p = ns) respectively). Neither lidocaine or riluzole changed the ECG intervals: there was no statistical significance between groups at time zero (just before ligation) for any ECG measure. During the course of the 3 hour period of the ischaemia R-R, and P-R intervals shortened slightly in control and riluzole groups (not significantly different from each other) but not in the lidocaine group (significantly different from control). QRS and QTc did not change appreciably in any group Riluzole reduced the degree of histopathological tissue damage across the infarct zone considerably more than did lidocaine.

**Conclusions:**

At the doses used, riluzole was at least as effective as lidocaine at reducing the number of episodes of ischaemic VT or VF in pigs, and much more effective at reducing the number of PVCs. We propose that this is related to the ability of riluzole to block cardiac persistent sodium current.

## Introduction

Sudden cardiac death (SCD) as a consequence of ischaemic arrhythmias is a major cause of mortality in the developed world, and, importantly, ventricular fibrillation-related mortality appears not to be declining despite a decline in the overall prevalence of coronary artery disease (CAD) [1]. A therapeutic approach to prevention of SCD would have a large impact on years of life lost. However, large SCD trials of antiarrhythmic drugs have had outcomes that have been either disappointing or, worse, showed an increase in mortality in the treatment group [2]. A potential novel target for cardiac ischaemic protection, the persistent sodium current (INaP), has recently gained increasing interest [3], [4]. Here we report results with riluzole, a potentially new cardiac protective agent which appears to act by blocking INaP, and compare its effectiveness for suppression of ischaemic arrhythmias with lidocaine.

Riluzole is currently approved for use in Man for amyotrophic lateral sclerosis (ALS), and it has also been shown to be protective against neuronal ischaemia in a variety of models [5], [6], [7]. This action of riluzole is generally thought to be due to its ability to block glutamate release and/or postsynaptic NMDA receptors [8], [9], although a variety of other mechanisms have been proposed, including inhibition of neuronal sodium channels [10]. We have recently shown that riluzole blocks the persistent sodium current (INaP) in single cardiomyocytes, is cardioprotective in isolated rat hearts, and prevents the rise in [Ca^2+^]_i_ induced in single myocytes by hypoxia and reoxygenation, all with more than a 10-fold greater potency than the block of the cardiac transient sodium current (INaT) [11] In that paper we also showed that, under normoxic conditions, riluzole did not affect the monophasic action potential in isolated rat hearts or the ECG intervals in pigs. We surmised that riluzole is protective against myocardial ischaemic and reperfusion damage via its action to block INaP.

However, results in myocardial cells, or tissue preparations, or small animal hearts, often do not predict effects on arrhythmogenesis in large animals, including man. In this study we were interested in the potential of riluzole to reduce the occurrence and/or severity of ischaemic arrhythmias (the leading clinical cause of sudden cardiac death). We compared riluzole to an established and well characterised drug, lidocaine. We show that riluzole reduces the occurrence of ischaemic arrhythmias in an *in vivo* pig model of acute myocardial infarction (AMI). We also show that riluzole reduces the extent of myocardial damage at both the gross and microscopic levels, and confirm that it has minimal impact on normal ECG intervals in the pig AMI model. Riluzole had greater anti-arrhythmic and anti-ischaemic potency than lidocaine in this model. We conclude that riluzole may be cardioprotective and antiarrhythmic in Phase 1b and Phase 2 ischaemia and possibly ischaemia-reperfusion settings, and that riluzole is more potent than lidocaine in these settings. We further suggest that these results shed light on the mechanisms underlying different phases of ischaemic arrhythmias.

## Methods

All procedures for these experiments had prior approval of the Animal Experimentation and Ethics Committee of the Australian National University: protocol numbers J.MB.38.08, J.MB.24.05, J.MB.09.03. The investigation conforms to the Guide for the Care and Use of Laboratory Animals published by the US National Institutes of Health (NIH Publication No. 85–23, revised 1996). All surgery was performed under anaesthesia, and all efforts were made to minimise suffering.

27 pigs (landrace or large white, either sex, 20–35 kg) were sedated with stresnil (Boehringer Ingelheim 1–2 mg/kg im) and anaesthetized with thiopentone sodium (Troy Laboratories 10–15 mg/kg iv) and isoflurane (Laser Animal Health 0.5 – 2%) in oxygen. Artificial ventilation (Ugo Basile) was maintained at 15 ml/kg/breath and 12 breaths per minute. Blood pressure (BP) and a lead II electrocardiogram (ECG) were monitored, digitised and recorded (Data Translation) for subsequent analysis. An intravenous normal saline drip was maintained for intra-operative hydration (control group: 10 animals) or lidocaine (Troy Laboratories) administration (lidocaine group: 11 animals, 2.5–12 mg/kg bolus plus 0.05–0.24 mg/kg/min iv continuous infusion). Riluzole (Sigma Pharmaceuticals) was administered intraperitoneally in 6 animals (riluzole group: 8 mg/kg in 2 ml propylene glycol). Myocardial infarctions were created by ligating the left anterior descending coronary arteries (LAD) mid-way along their lengths approximately 50 min after the commencement of saline, lidocaine or riluzole administration. Regional ischaemia was confirmed by visual inspection for cyanosis and dyskinetic wall motion [12]. Each experiment was terminated 3 hours after ligation.

Measurements of the overall length of each LAD and the corresponding length of the ischaemic portion were made in each animal to ensure that the extent of induced myocardial damage was comparable between the control, lidocaine and riluzole groups. Area at risk and the size of the infarcted zone were not formally measured, although gross pathological changes were assessed visually.

Data collection included counts of the number of premature contractions, including PVCs and those in alternating rhythms such as bigeminy and trigeminy, during the first three hours post infarction. Data collection also included counts of the number of runs of non-sustained arrhythmias (episodes of spontaneous ventricular tachycardia (VT) or ventricular fibrillation (VF) lasting for more than 1 s but self-terminating within 15 s of commencement) and counts of the number of episodes of sustained VT or VF (lasting for 15 s or more and requiring cardioversion or defibrillation). Data analysis comprised comparing the number of arrhythmias both by phase and by cumulative grouping.

Gross pathology was performed by a visual comparison of the epicardial surface of each of the hearts in each of the groups post fixation in 10% buffered formalin. Microscopic pathology was performed by sampling full thickness sections of the myocardium from the ligation site to the apex. Tissue was fixed in 10% neutral buffered formalin and processed using routine laboratory methods into wax blocks; sections were stained with hematoxylin and eosin (H&E).

Statistical analysis was performed using Graphpad Prism. Overall statistical significance was assessed using ANOVA, followed by inter-group comparisons using t-tests if ANOVA showed significance. Data is presented as mean +/− SEM. Significance was taken at p<0.05 and was determined using Student's t-tests.

## Results

The positions of the ligations along the LAD, and hence the proportions of ischaemic LAD to the overall length of the LAD, averaged 52 +/−1% in the control group, 59 +/−2% in the lidocaine group, and 57 +/−2% in the riluzole group (not significantly different).

Over the 3 hr post ligation period, all animals developed single and multiple PVCs (doublets and triplets), though only 60% of control animals, 45% of lidocaine animals and 33% of riluzole animals developed bigeminy or trigeminy. While 90% of control animals developed non-sustained VT or VF, only 36% of lidocaine animals and 50% of riluzole animals did. While 100% of control animals developed sustained VT or VF, only 55% of lidocaine animals and 50% of riluzole animals did.

**Figure 1:**
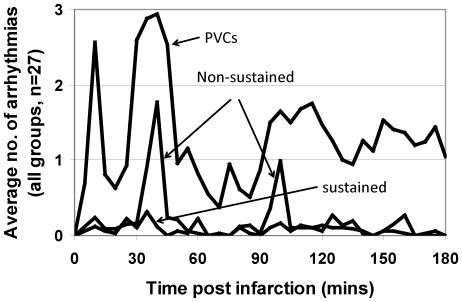
Time course of arrhythmias. Graphs of the average number of arrhythmias per animal in each 5 minute time period over the duration of each experiment for each of the three groups of arrhythmias: PVCs (including single and multiple PVCs and alternating rhythms such as bigeminy and trigeminy), non-sustained ventricular tachycardia and ventricular fibrillation (lasting for more than 1 s but self-terminating within 15 s of commencement), and sustained ventricular tachycardia and ventricular fibrillation (lasting for more than 15 s and requiring cardioversion or defibrillation). Note that the number of PVCs shown is 1/10^th^ of the actual number of PVCs recorded (to enable display on the same graph). The periods of peak arrhythmia activity can be seen to extend between 0 and 20 mins, 20 and 50 mins, and 50 and 180 mins, corresponding to Phase 1a, Phase 1b and Phase 2 arrhythmias respectively.

Figure 1 shows the average incidence of arrhythmias for all animals across the PVC, non-sustained and sustained arrhythmia classifications. Consistent with published data [13], there appeared to be three distinct phases of high arrhythmia incidence: Phase 1a arrhythmias occurred in the period from 0 to 20 minutes post ligation, Phase 1b arrhythmias occurred in the period from 20 to 50 minutes post ligation, and Phase 2 occurred in the period from 50 to 180 minutes [14], [15], [16].

**Figure 2:**
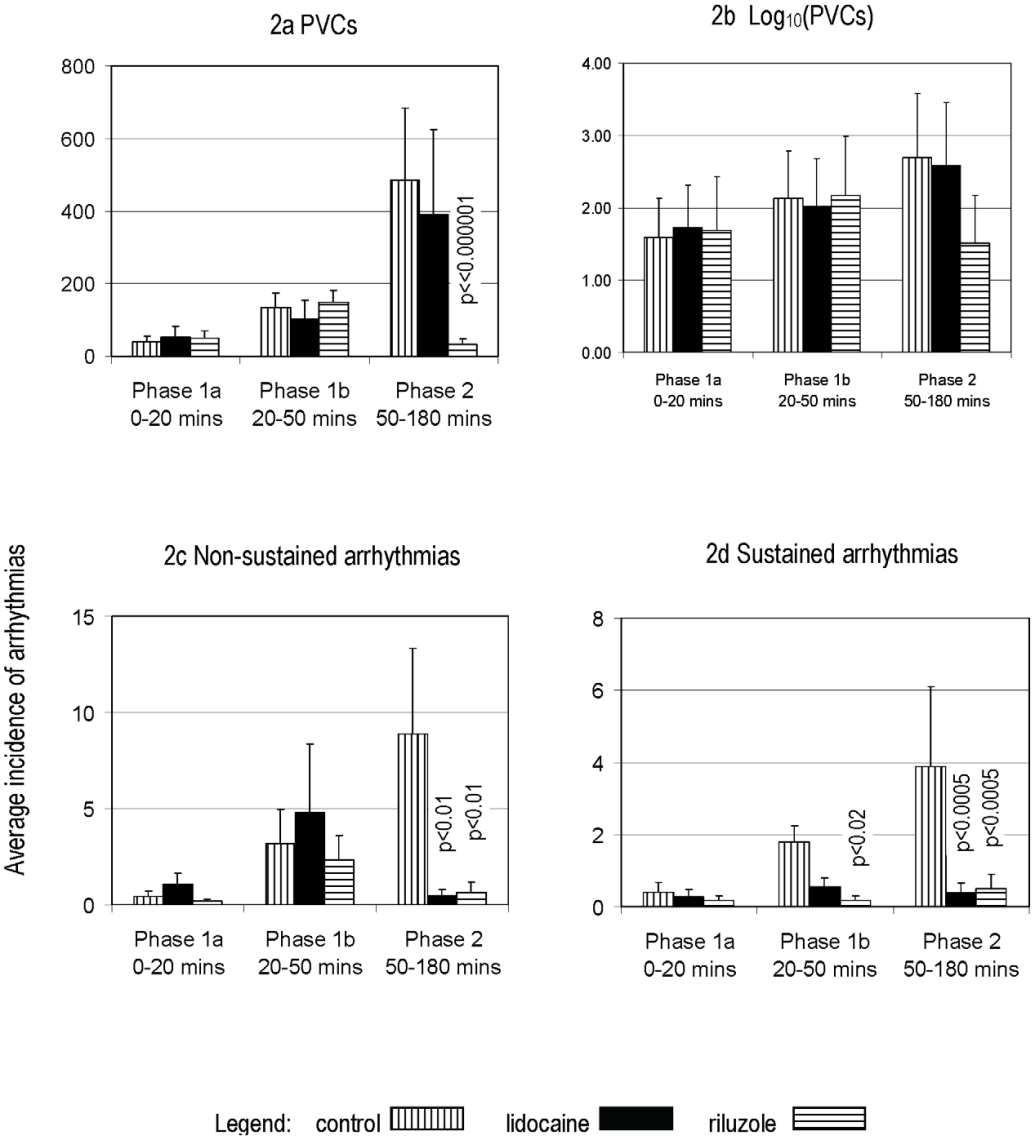
Average number of arrhythmias by phase. Comparative graphs showing the average number of each arrhythmia type by phase for the control, lidocaine and riluzole groups. Note that riluzole and lidocaine significantly reduced the number of Phase 2 non-sustained and sustained arrhythmias. Note also that riluzole and lidocaine reduced the number of Phase 1b sustained arrhythmias but that this was not significant for lidocaine. In addition, riluzole significantly reduced the number of Phase 2 PVCs whereas lidocaine had minimal effect.

Compared with controls, riluzole significantly reduced the incidence of all types of arrhythmias during Phase 2 (Figure 2, and table 1). Riluzole also significantly reduced the incidence of sustained arrhythmias during Phase 1b but did not reduce the incidence of PVCs or non-sustained arrhythmias during Phase 1b or any of the arrhythmias in Phase 1a. Compared with controls, lidocaine significantly reduced the incidence of non-sustained and sustained arrhythmias during Phase 2. In marked contrast to riluzole, lidocaine did not reduce the incidence of PVCs in phase 2. While lidocaine did reduce the incidence of sustained arrhythmias during Phase 1b, the reduction did not reach statistical significance. Conversely, lidocaine appeared to increase the incidence of non-sustained arrhythmias during phase 1b, although this increase did not reach statistical significance. Lidocaine did not reduce the incidence of PVCs during Phase 1b and did not reduce the incidence of any arrhythmia types during Phase 1a.

**Table 1:** Average number of arrhythmias per animal for each group in each of the phases. “n” refers to the number of animals in each group.

		CONTROL (n = 10)		LIDOCAINE (n = 11)		RILUZOLE (n = 6)	
		mean	SEM	mean	SEM	mean	SEM
PVCs	Phase 1a0–20 mins	38.90	15.57	53.00	28.68	49.33	21.11
	Phase 1b20–50 mins	134.10	39.08	102.45	50.96	148.67	31.84
	Phase 250–180 mins	484.60	199.86	390.09	234.09	32.50	13.32
	**TOTAL**	**657.60**	**190.19**	**545.55**	**278.73**	**230.50**	**78.96**
Non-sustained arrhythmias	Phase 1a0–20 mins	0.40	0.31	1.09	0.54	0.17	0.13
	Phase 1b20–50 mins	3.20	1.74	4.82	3.55	2.33	1.24
	Phase 250–180 mins	8.90	4.41	0.45	0.33	0.67	0.52
	**TOTAL**	**12.50**	**4.15**	**6.36**	**3.77**	**3.17**	**2.26**
Sustained arrhythmias	Phase 1a0–20 mins	0.40	0.27	0.27	0.20	0.17	0.13
	Phase 1b20–50 mins	1.80	0.44	0.55	0.26	0.17	0.13
	Phase 250–180 mins	3.90	2.21	0.36	0.29	0.50	0.39
	**TOTAL**	**6.10**	**2.61**	**1.18**	**0.40**	**0.83**	**0.48**

### PVCs

**Figure 3:**
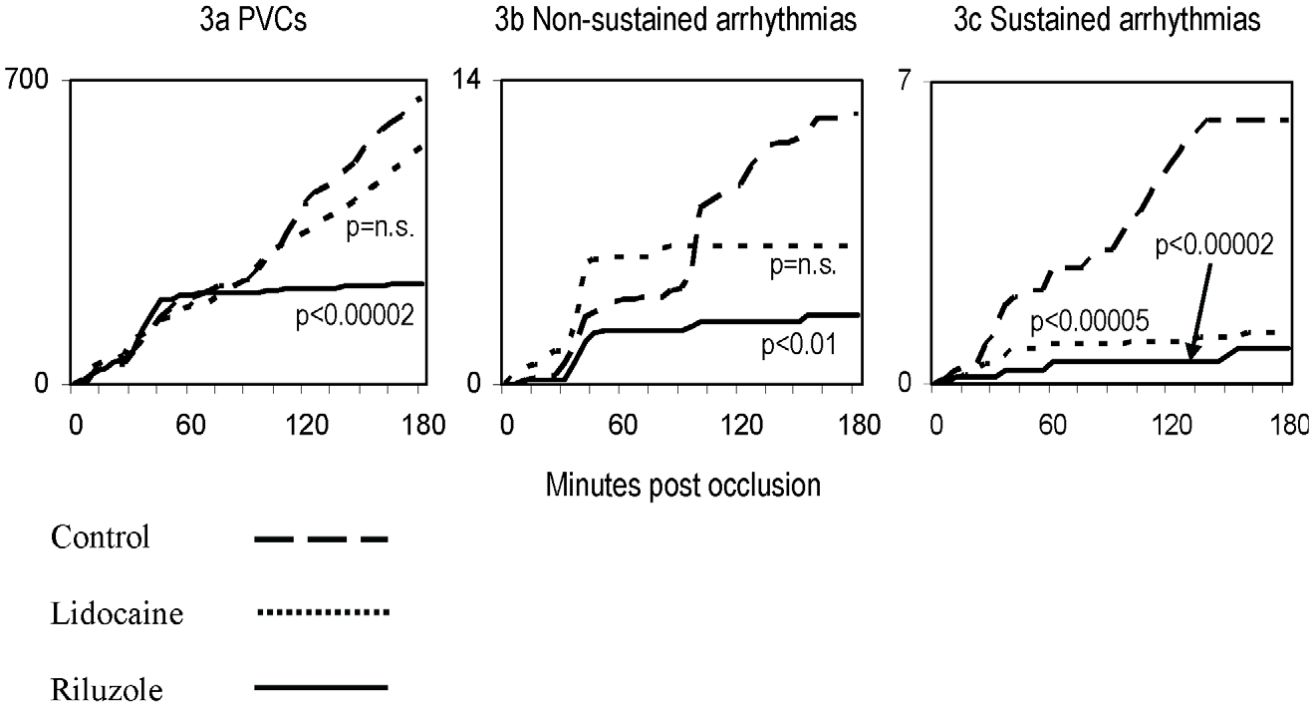
Cumulative incidence of arrhythmias. Comparative graphs showing reductions in the cumulative average number of arrhythmias per animal by arrhythmia types for control, lidocaine and riluzole groups of animals. Note the significant reduction in the number of each type of arrhythmia by riluzole as compared with lidocaine and control (note different scales on y axes).

Cumulative data showed that for the first 45 minutes following coronary artery ligation, there was a steady increase in the number of PVCs in each of the control, lidocaine and riluzole groups. However, during the 50 to 180 minute period post ligation, there were very few PVCs in the riluzole group while the incidence of PVCs in the control and lidocaine groups continued to rise steadily (Figure 3a). The difference between the cumulative growth in the number of PVCs in the control and riluzole groups was highly significant, at p<0.00002. The difference between the control and lidocaine groups was not significant.

### Non-sustained arrhythmias

While the growth in the cumulative number of non-sustained arrhythmias levelled off in both the lidocaine and riluzole groups after 45 minutes post ligation, there were many non-sustained arrhythmias in the 35 to 45 minute post ligation period in the lidocaine group which caused there to be no significant difference between the cumulative number of non-sustained arrhythmias in the control and lidocaine groups (Figure 3b). In contrast, the difference between the cumulative number of arrhythmias in the control and riluzole groups was significant (p<0.01). The growth in the cumulative number of non-sustained arrhythmias in the riluzole group was about half that of the lidocaine group.

### Sustained arrhythmias

The growth in the cumulative number of sustained arrhythmias was significantly lower in both the lidocaine (p<0.00005) and riluzole (p<0.00002) groups than in the control group (Figure 3c, and table 1). As with the growth in the cumulative number of non-sustained arrhythmias, the growth in the cumulative number of sustained arrhythmias in the riluzole group was about half that of the lidocaine group.

### ECG intervals

**Figure 4:**
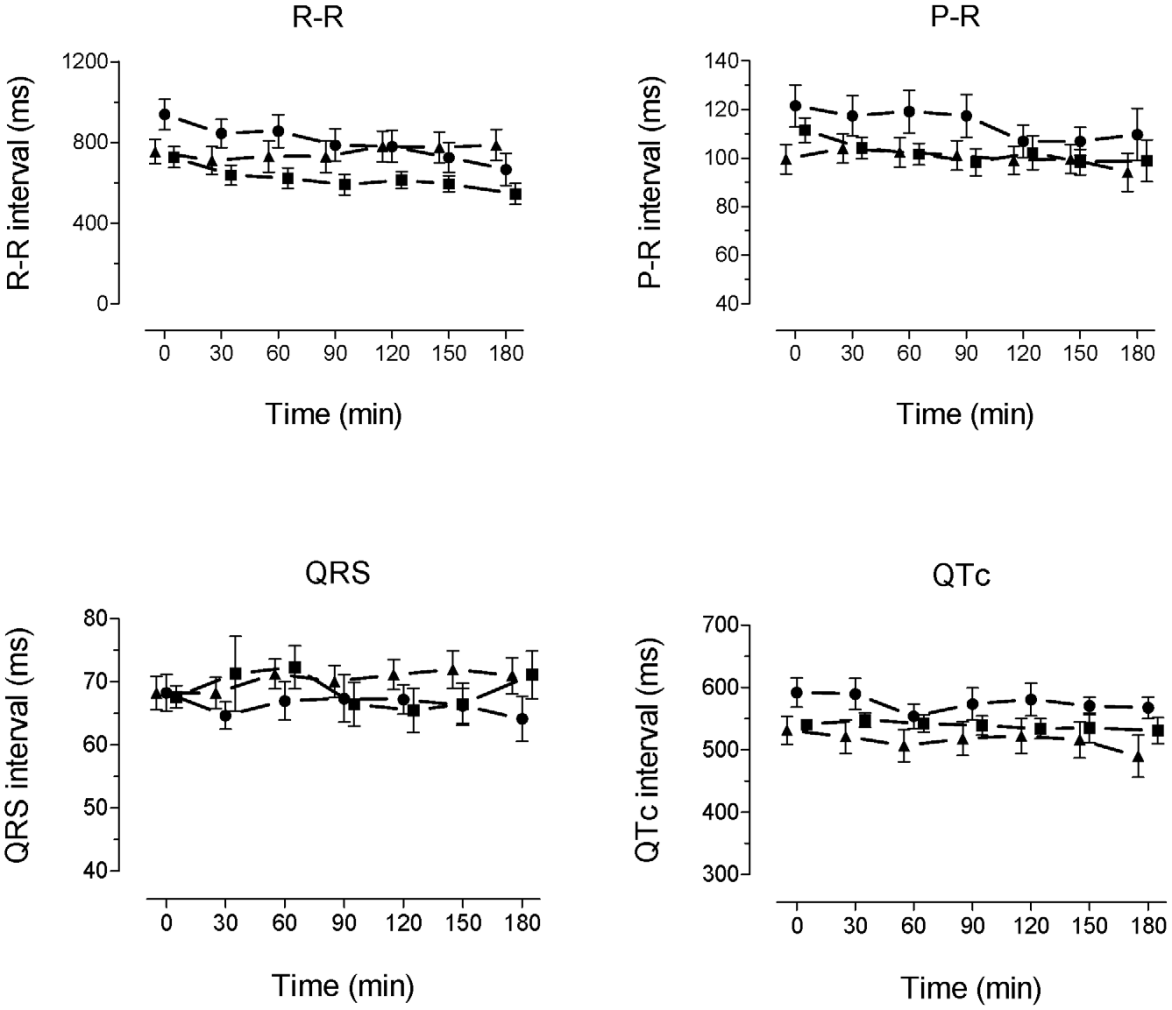
Time course of ECG intervals. Plots of R-R, P-R, QRS and QTc intervals during the experiment. Data are shown for 30, 60, 90, 120, 150 and 180 min after ligation (points are displaced for clarity). Symbols show mean +/− SEM. Control  =  filled circles, riluzole  =  filled squares, lidocaine  =  filled triangles.

ECG intervals were not significantly different between the groups after drug administration and before ligation. During the course of the 3 hour period of ischaemia R-R intervals shortened slightly in control and riluzole treated animals (p <0.001) but this was not seen in the lidocaine treated animals. Similar results were seen with P-R interval; a slight shortening in control and riluzole treated animals which was not seen in the lidocaine group QRS and QTc did not change appreciably during the course of the 3 hour ischaemia in any group. (figure 4)

### Arrhythmogenesis

**Figure 5:**
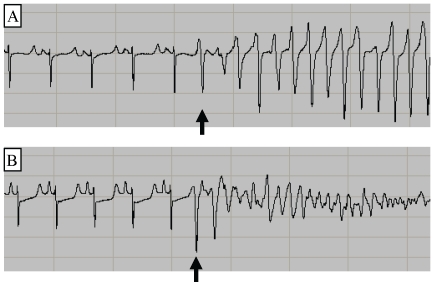
PVC-initiation of arrhythmias. Panel A shows a PVC initiated self-terminating ventricular tachycardia while panel B shows a PVC-initiated ventricular fibrillation. The arrows show the arrhythmia-initiating PVCs falling on the previous T-wave.

Most sustained and non-sustained arrhythmias were initiated by an R-wave falling within the vulnerable period of the preceding T-wave. Example traces are shown for a non-sustained arrhythmia in figure 5A and for a sustained arrhythmia in figure 5B.

### Histology

**Figure 6:**
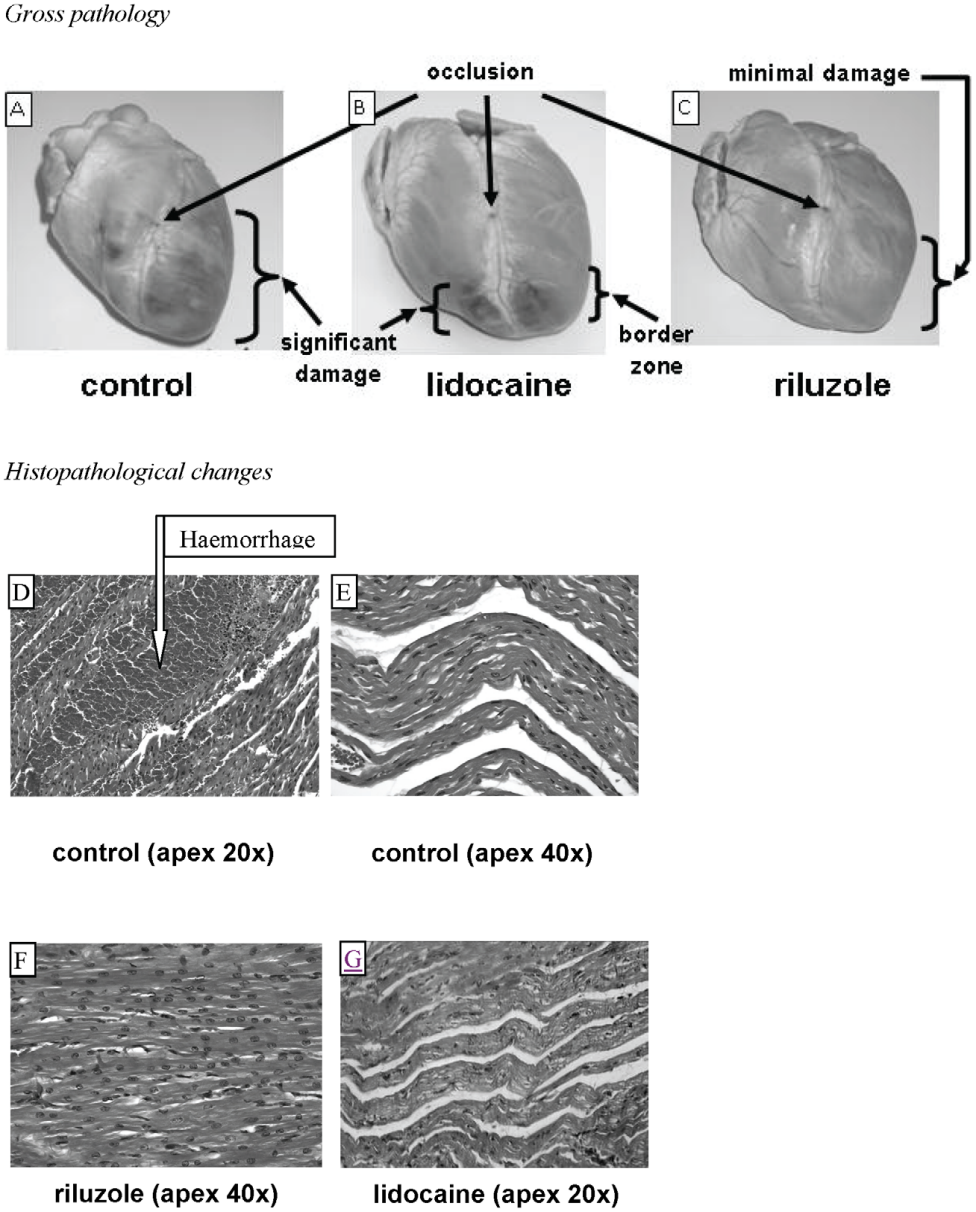
Pathologic changes. *Upper panels:* Examples of the gross pathologic damage in the control (panel A), lidocaine (panel B) and riluzole (panel C) groups. Note the shadow representative of the border zone in the lidocaine group and the minimal damage in the riluzole group. *Lower panels:* Examples of microscopic myocardial damage. Panel D shows haemorrhage and oedema in a control heart at low magnification. Panel E shows extensive oedema in a control heart at high magnification. Panel F shows minimal haemorrhage or oedema in a riluzole heart at high magnification. Panel G shows moderate oedema in a lidocaine heart at low magnification.

Gross examination of the hearts showed significant damage of the anterior walls of both the left and right ventricular surfaces extending from the ligation to the apex on all control hearts (Figure 6A). In contrast, the hearts from the lidocaine group showed smaller areas of damage towards the anterior apical region of both ventricles (Figure 6B) while in the riluzole group, there was minimal gross pathological damage evident (Figure 6C).

Microscopically, the anterior apical regions of the control hearts showed extensive interstitial haemorrhage and oedema (Figure 6D and 6E). In contrast, the anterior apical regions of the riluzole hearts showed minimal oedema or haemorrhage (Figure 6F). The extent of haemorrhage and oedema in the anterior apical region of the lidocaine-treated hearts was more similar to that of the control hearts (Figure 6G). Contraction band necrosis was most evident in the control group. There was no inflammatory cell infiltrate.

## Discussion

### Phases of ischaemic arrhythmias

Reports of arrhythmias occurring in phases following an AMI date back to 1950 [17]; the phases having been classified as Phase 1, associated with the period of reversible injury [1] and Phase 2, which commences during the early inflammatory stage of infarct evolution [18] and is defined by the establishment of myocardial necrosis [1]. Further studies found that Phase 1 arrhythmias comprised two distinct sub-classifications, now known as Phase 1a and Phase 1b [14], [19], [20] with Phase 1b defined as heralding the onset of irreversible myocardial damage [21]. Phase 1a arrhythmias are generally considered to commence between 0 and 5 minutes post coronary artery occlusion [13], [22], [23] and terminate between 3 and 20 minutes post occlusion [24], [25], [26]. Phase 1b arrhythmias are generally defined as commencing between 5 and 20.5 minutes [13], [16], [27] and terminating between 18 and 90 minutes post occlusion [24], [28], [29], [30]. Phase 2 arrhythmias commence after phase 1b [16].

Consistent with this, in this study in *in vivo* pigs, we found that Phase 1a spanned from 0 to 20 minutes, Phase 1b spanned from 20 to 50 minutes, and Phase 2 spanned from 50 to 180 minutes (the time of termination of each experiment) after coronary artery occlusion (figure 1). We also found that neither lidocaine nor riluzole significantly affected the incidence of any of the types of Phase 1a arrhythmias but that both reduced the incidence of sustained arrhythmias during Phase 1b, although this was significant at P<0.02 only for riluzole. Most notably, riluzole significantly reduced the incidence of Phase 2 arrhythmias irrespective of the type of arrhythmia (PVCs, doublets, triplets, bigeminy, trigeminy, non-sustained VT, non-sustained VF, sustained VT and sustained VF).

### Mechanism of action

We have previously shown that riluzole blocks myocardial INaP and reduces the rise in intracellular [Ca^2+^] resulting from hypoxia [31] and we now show here that riluzole significantly reduces the extent of myocardial damage at both the gross and microscopic levels following 3 hours of ischaemia. Similarly, it has been shown that lidocaine blocks INaP [32] and in these experiments lidocaine also reduced the extent of myocardial damage, although not as well as did riluzole. We therefore propose that INaP blockers, and in particular riluzole, can reduce the extent of myocardial damage as well as the incidence of Phase 2 arrhythmias and Phase 1b sustained arrhythmias.

### Myocardial damage

Saint et al [33] showed that, under normal conditions, INaP allows a Na^+^ influx with an amplitude of around 0.5% of that of the transient sodium current. However, under hypoxic conditions such as ischaemia, INaP is enhanced by about a factor of 3 or 4 [34]. Because INaP does not inactivate, it can potentially cause a large influx of Na^+^ and the consequent rise in [Na^+^]i can produce a slowing or reversal of the myocardial sodium-calcium exchanger (NCX) [35]. The subsequent overloading of cells with Ca^2+^ causes myocardial damage, loss of gap junctions, and impaired cell coupling [36]. Hence, it appears that lidocaine and riluzole, both myocardial INaP blockers, significantly reduced the extent of myocardial damage from ischaemia by reducing the [Na^+^]_i_ overload and consequent sequence of events leading to [Ca^2+^]_i_ loading and cell damage.

### Arrhythmias

There are three potential mechanisms by which arrhythmias can be maintained in ventricular myocardium - re-entrant, triggered activity, and abnormal automaticity. Re-entrant arrhythmias require unidirectional conduction, conduction block, and a region of slow conduction such that the cycle length of the arrhythmia is longer than the longest refractory period in the circuit [37]. All these factors can result from myocardial damage and in particular the loss of gap junctions, impaired cell coupling and myocardial heterogeneity which occur during Phase 1b and Phase 2 [36]. Therefore, given the coincident timing of Phase 1b and Phase 2 arrhythmias seen in this study with the development of myocardial damage and the substrate required for re-entrant arrhythmias, it would appear that riluzole and lidocaine reduced the volume and/or heterogeneity of myocardial damage and in so-doing, reduced the damage to an amount too small and/or too homogenous to sustain re-entrant arrhythmias. It appears that riluzole is more potent than lidocaine in reducing myocardial damage and the incidence of re-entrant arrhythmias, possibly because the selectivity for block of INaP over INaT is higher for riluzole than for lidocaine. Alternatively, given that lidocaine was less effective in reducing myocardial damage than riluzole, its effect to reduce arrhythmias may have been due to its class Ib action.

While re-entrant arrhythmias require a suitable substrate in order to be sustained, they also require a stimulus for initiation. As exemplified in Figures 5A and 5B, many of the non-sustained and sustained arrhythmias were initiated by single PVCs falling in the middle of the preceding T-wave; this portion of the T-wave corresponding to the vulnerable period for R-on-T initiation of arrhythmias [38], [39]. While there is the possibility that arrhythmias in this study were initiated by an automatic focus, the coincident timing with the T-wave makes this outcome unlikely and it is far more likely that most of the arrhythmias in this study were initiated by EADs.

We note some potentially important differences between lidocaine and riluzole. 1. Riluzole blocked PVCs during phase 2, whereas lidocaine did not. We suggest that this indicates that riluzole is more effective at preventing the calcium overload contributing to DADs. 2. There was a trend for lidocaine to be proarrhythmic for non-sustained arrhythmias during Phase 1b. This may indicate that lidocaine at this time is exacerbating electrical heterogeneity because of its effects on conduction velocity and threshold, via block of INaT, while riluzole does not produce these effects. 3. Riluzole did not change the ECG intervals before ligation at anti-arrhythmic doses and did not affect the ECG intervals during the 3 hour period of ischaemia. We suggest that these differences are due to the more selective block of INaP over INaT by riluzole, as compared to lidocaine. Our results suggest that the Phase 2 arrhythmias and the Phase 1b sustained arrhythmias observed in this study were re-entrant arrhythmias initiated by triggered activity which was DAD driven. We hypothesise that, by blocking myocardial INaP, riluzole and lidocaine reduced the rise in intracellular [Ca^2+^] required to cause triggered activity and in so-doing, reduced the stimulus for initiating the Phase 2 arrhythmias and the Phase 1b non-sustained arrhythmias as well as the size of the substrate required for the maintenance of re-entrant arrhythmias. We further propose that riluzole was more effective than lidocaine in reducing the extent of myocardial damage and the incidence of Phase 2 PVCs and Phase 1b non-sustained arrhythmias because of their different actions on cardiomyocytes and conduction.

The results demonstrate a potent effect of riluzole against ischaemia-induced arrhythmias and ischaemic damage. We suggest that these actions are due to action at a potential novel target, the cardiac persistent sodium current.

## References

[1] Clements-Jewery H, Hearse DJ, Curtis MJ (2005). Phase 2 ventricular arrhythmias in acute myocardial infarction: a neglected target for therapeutic antiarrhythmic drug development and for safety pharmacology evaluation.. Br J Pharmacol.

[2] Pratt CM, Camm AJ, Cooper W, Friedman PL, MacNeil DJ (1998). Mortality in the Survival With ORal D-sotalol (SWORD) trial: why did patients die?. Am J Cardiol.

[3] Saint DA (2008). The cardiac persistent sodium current: an appealing therapeutic target?. Br J Pharmacol.

[4] Vacher B, Pignier C, Letienne R, Verscheure Y, Le Grand B (2009). F 15845 inhibits persistent sodium current in the heart and prevents angina in animal models.. Br J Pharmacol.

[5] Bae HJ, Lee YS, Kang DW, Koo JS, Yoon BW (2000). Neuroprotective effect of low dose riluzole in gerbil model of transient global ischemia.. Neurosci Lett.

[6] Baptiste DC, Fehlings MG (2006). Pharmacological approaches to repair the injured spinal cord.. J Neurotrauma.

[7] Heurteaux C, Laigle C, Blondeau N, Jarretou G, Lazdunski M (2006). Alpha-linolenic acid and riluzole treatment confer cerebral protection and improve survival after focal brain ischemia.. Neuroscience.

[8] Bryson HM, Fulton B, Benfield P (1996). Riluzole. A review of its pharmacodynamic and pharmacokinetic properties and therapeutic potential in amyotrophic lateral sclerosis.. Drugs.

[9] Lamanauskas N, Nistri A (2008). Riluzole blocks persistent Na+ and Ca2+ currents and modulates release of glutamate via presynaptic NMDA receptors on neonatal rat hypoglossal motoneurons in vitro.. Eur J Neurosci.

[10] Ates O, Cayli SR, Gurses I, Turkoz Y, Tarim O (2007). Comparative neuroprotective effect of sodium channel blockers after experimental spinal cord injury.. J Clin Neurosci.

[11] Weiss S, Benoist D, White E, Teng W, Saint DA (2010). Riluzole protects against cardiac ischaemia and reperfusion damage via block of the persistent sodium current.. British Journal of Pharmacology.

[12] Walker MJ, Curtis MJ, Hearse DJ, Campbell RW, Janse MJ (1988). The Lambeth Conventions: guidelines for the study of arrhythmias in ischaemia infarction, and reperfusion.. Cardiovasc Res.

[13] Cascio WE, Yang H, Muller-Borer BJ, Johnson TA (2005). Ischemia-induced arrhythmia: the role of connexins, gap junctions, and attendant changes in impulse propagation.. J Electrocardiol.

[14] Curtis MJ (1998). Characterisation, utilisation and clinical relevance of isolated perfused heart models of ischaemia-induced ventricular fibrillation.. Cardiovasc Res.

[15] Meesmann W, Parrat JR (1982). Early arrhythmias and primary ventricular fibrillation after acute myocardial ischaemia in relation to pre-existing coronary collaterals.. Early Arrhythmias Resulting from Myocardial Ischaemia.

[16] Pugsley MK, Ries CR, Guppy LJ, Harvie CJ, Walker MJ (1995). Effects of anipamil, a long acting analog of verapamil, in pigs subjected to myocardial ischemia.. Life Sci.

[17] Harris AS (1950). Delayed development of ventricular ectopic rhythms following experimental coronary occlusion.. Circulation.

[18] Clements-Jewery H, Hearse DJ, Curtis MJ (2007). Neutrophil ablation with anti-serum does not protect against phase 2 ventricular arrhythmias in anaesthetised rats with myocardial infarction.. Cardiovasc Res.

[19] Bernus O, Zemlin CW, Zaritsky RM, Mironov SF, Pertsov AM (2005). Alternating conduction in the ischaemic border zone as precursor of reentrant arrhythmias: a simulation study.. Europace.

[20] Cheema AN, Sheu K, Parker M, Kadish AH, Goldberger JJ (1998). Nonsustained ventricular tachycardia in the setting of acute myocardial infarction: tachycardia characteristics and their prognostic implications.. Circulation.

[21] Remme CA, Wilde AA (2000). KATP channel openers, myocardial ischemia, and arrhythmias–should the electrophysiologist worry?. Cardiovasc Drugs Ther.

[22] Wainwright CL, Parratt JR (1988). An antiarrhythmic effect of adenosine during myocardial ischaemia and reperfusion.. Eur J Pharmacol.

[23] Yan GX, Joshi A, Guo D, Hlaing T, Martin J (2004). Phase 2 reentry as a trigger to initiate ventricular fibrillation during early acute myocardial ischemia.. Circulation.

[24] Barrett TD, MacLeod BA, Walker MJ (1997). A model of myocardial ischemia for the simultaneous assessment of electrophysiological changes and arrhythmias in intact rabbits.. J Pharmacol Toxicol Methods.

[25] Dilly SG, Lab MJ (1988). Electrophysiological alternans and restitution during acute regional ischaemia in myocardium of anaesthetized pig.. J Physiol.

[26] Sedlis SP (1992). Mechanisms of ventricular arrhythmias in acute ischemia and reperfusion.. Cardiovasc Clin.

[27] Gumina RJ, Daemmgen J, Gross GJ (2000). Inhibition of the Na(+)/H(+) exchanger attenuates phase 1b ischemic arrhythmias and reperfusion-induced ventricular fibrillation.. Eur J Pharmacol.

[28] Russell DC, Lawrie JS, Riemersma RA, Oliver MF (1984). Mechanisms of phase 1a and 1b early ventricular arrhythmias during acute myocardial ischemia in the dog.. Am J Cardiol.

[29] Parker KK, Lavelle JA, Taylor LK, Wang Z, Hansen DE (2004). Stretch-induced ventricular arrhythmias during acute ischemia and reperfusion.. J Appl Physiol.

[30] Clements-Jewery H, Hearse DJ, Curtis MJ (2002). Independent contribution of catecholamines to arrhythmogenesis during evolving infarction in the isolated rat heart.. Br J Pharmacol.

[31] Weiss SM, Benoist D, White E, Saint DA (2010). Riluzole protects against cardiac ischaemia and reperfusion damage via block of the persistent sodium current.. British Journal of Pharmacology (in press).

[32] Ju YK, Saint DA, Gage PW (1992). Effects of lignocaine and quinidine on the persistent sodium current in rat ventricular myocytes.. Br J Pharmacol.

[33] Saint DA, Ju YK, Gage PW (1992). A persistent sodium current in rat ventricular myocytes.. J Physiol.

[34] Ju YK, Saint DA, Gage PW (1996). Hypoxia increases persistent sodium current in rat ventricular myocytes.. J Physiol.

[35] Satoh H, Mukai M, Urushida T, Katoh H, Terada H (2003). Importance of Ca2+ influx by Na+/Ca2+ exchange under normal and sodium-loaded conditions in mammalian ventricles.. Mol Cell Biochem.

[36] Dekker LR, Rademaker H, Vermeulen JT, Opthof T, Coronel R (1998). Cellular uncoupling during ischemia in hypertrophied and failing rabbit ventricular myocardium: effects of preconditioning.. Circulation.

[37] Riley MP, Marchlinski FE (2008). ECG clues for diagnosing ventricular tachycardia mechanism.. J Cardiovasc Electrophysiol.

[38] Weiss SM, Einstein R, McCulloch RM (1993). A new method of fibrillation induction for implantable cardioverter-defibrillator testing.. Journal of Clinical Engineering.

[39] Swerdlow CD, Shehata M, Chen PS (2007). Using the upper limit of vulnerability to assess defibrillation efficacy at implantation of ICDs.. Pacing Clin Electrophysiol.

